# Gene expression in the mixotrophic prymnesiophyte, *Prymnesium parvum*, responds to prey availability

**DOI:** 10.3389/fmicb.2015.00319

**Published:** 2015-04-20

**Authors:** Zhenfeng Liu, Adriane C. Jones, Victoria Campbell, K. David Hambright, Karla B. Heidelberg, David A. Caron

**Affiliations:** ^1^Department of Biological Sciences, University of Southern CaliforniaLos Angeles, CA, USA; ^2^Program in Ecology and Evolutionary Biology, Department of Biology, University of OklahomaNorman, OK, USA

**Keywords:** *Prymnesium parvum*, transcriptomics, gene expression, mixotrophic protist, golden algae, harmful algae

## Abstract

The mixotrophic prymnesiophyte, *Prymnesium parvum*, is a widely distributed alga with significant ecological importance. It produces toxins and can form ecosystem disruptive blooms that result in fish kills and changes in planktonic food web structure. However, the relationship between *P. parvum* and its prey on the molecular level is poorly understood. In this study, we used RNA-Seq technology to study changes in gene transcription of *P. parvum* in three treatments with different microbial populations available as potential prey: axenic *P. parvum* (no prey), bacterized *P. paruvm*, and axenic *P. parvum* with ciliates added as prey. Thousands of genes were differentially expressed among the three treatments. Most notably, transcriptome data indicated that *P. parvum* obtained organic carbon, including fatty acids, from both bacteria and ciliate prey for energy and cellular building blocks. The data also suggested that different prey provided *P. parvum* with macro- and micro-nutrients, namely organic nitrogen in the form of amino acids from ciliates, and iron from bacteria. However, both transcriptomic data and growth experiments indicated that *P. parvum* did not grow faster in the presence of prey despite the gains in nutrients, although algal abundances attained in culture were slightly greater in the presence of prey. The relationship between phototrophy, heterotrophy and growth of *P. parvum* is discussed.

## Introduction

*Prymnesium parvum* is a globally distributed prymnesiophyte alga of considerable ecological importance. *P. parvum* occurs in ecosystems across a wide range of salinities and environmental conditions (Guo et al., 1996; Barkoh and Fries, 2010; Johnsen et al., 2010), possesses a complex life history including haploid and diploid stages (Larsen, 1999), and exhibits mixtrophic abilities (Tillmann, 2003). The combined photosynthetic and heterotrophic capabilities of *P. parvum* enable the alga to form Ecosystem Disruptive Algal Blooms (EDABS) (Sunda et al., 2006). The disruptive nature of the alga to the ecosystems in which it blooms has implicated *P. parvum* as a cause of significant economic loss to the aquaculture industry worldwide (Barkoh and Fries, 2010).

Commonly referred to as the “golden alga,” *P. parvum* has appeared and attained bloom abundances in several waterways of the southwestern United States during the last two decades (Roelke et al., 2011; Hambright et al., 2015). Genetic analyses of *P. parvum* populations have indicated a relatively recent appearance of this species in the region, perhaps a consequence of a recent introduction of a strain from Europe, and subsequent range expansion (Lutz-Carrillo et al., 2010; Hambright et al., 2015). The alga has resulted in ecosystem disturbances ranging from changes in plankton food web structure to massive fish kills (Southard et al., 2010; Jones et al., 2013). Various reports have noted that substances produced by *P. parvum* can immobilize a range of co-occurring species and thereby aid in the capture of prey that are often considerably larger than the alga (Henrikson et al., 2010; Remmel and Hambright, 2012). These substances may also act as predator deterrents for potential consumers or allelopathic agents retarding the growth of competing phytoplankton (Schmidt and Hansen, 2001; Fistarol et al., 2003; Granéli and Salomon, 2010; Remmel et al., 2011).

*P. parvum* exhibits robust phototrophic growth in culture, a characteristic that is not always common for mixotrophic microalgae (Jones, 1997). Aside from a requirement for the vitamins thiamine (B_1_) and cobalamin (B_12_) (McLaughlin, 1958; Rahat and Reich, 1963), the alga is capable of growth on inorganic medium. Maximal growth rates of 0.6–0.8 d^−1^ have been reported for *Prymnesium* species in axenic culture (Granéli et al., 2012; Hambright et al., 2014). Conversely, growth of *P. parvum* in the dark is not supported by the presence of prey, and has only been achieved when the growth medium has been supplemented with high concentrations of specific organic compounds (most notably glycerol) (Rahat and Jahn, 1965). Moreover, growth rates in the dark in the presence of high concentrations of glycerol that have been reported for this species are considerably slower than growth in the light (Rahat and Jahn, 1965).

Despite a very limited ability to grow exclusively heterotrophically, *P. parvum* grown in the light displays clear predatory behaviors, and has been shown to be predatory on a number of types of microbes including bacteria, protists and a variety of metazoan zooplankton (Tillmann, 2003; Sopanen et al., 2006; Brooks et al., 2010). It has been suggested that up to 78% of the cellular nitrogen and 45% of the cellular phosphorus may be acquired via predation (Carvalho and Granéli, 2010). The heterotrophic capabilities of the alga include the production of a suite of toxins that exhibit hemolytic, ichthyotoxic, cytolytic, and potentially neurotoxic activities (Edvardsen and Paasche, 1998; Barkoh and Fries, 2010; Henrikson et al., 2010; Bertin et al., 2012; Granéli et al., 2012), and toxin characterization is an area of active research (Schug et al., 2010; Weissbach and Legrand, 2012; Manning and La Claire, 2013).

An alternative approach to understanding the physiological response of microbial eukaryotes to environmental factors has recently been enabled through genomic and transcriptomic analysis (Bik and Thomas, 2012; Murray et al., 2012). The latter approach has proven particularly fruitful for microbial eukaryotes in recent years (Keeling et al., 2014) because the larger genomes of many of these species relative to bacteria and archaea incur much greater sequencing costs and difficulties with genome assembly. The genome of *P. parvum* has not yet been sequenced, but a few studies examining gene expression in the species using a variety of methods have been conducted. La Claire (2006) established the first EST database for *P. parvum* and examined the most frequently detected genes. Beszteri et al. (2012) used the same approach to study *P. parvum* gene expression under N or P limitations. Freitag et al. (2011) correlated the expression of certain polyketide synthase genes (genes assumed to play a role in toxin production) by qPCR with toxicity of the alga when cultures were exposed to physiological shock. Koid et al. (2014) compared the gene contents of *P. parvum* and other three prymnesiophytes on a whole transcriptome level. These studies have provided baseline information on gene expression by the prymnesiophyte.

We conducted a transcriptomic analysis of *P. parvum* using RNA-Seq designed to specifically examine the effect of the presence or absence of prey on gene expression. Gene expression of the alga was analyzed when grown in laboratory culture for 2–3 weeks in three situations; (1) axenically, (2) in the presence of a mixed bacterial assemblage (potential prey and/or symbiotic interactions), and (3) grown in the presence of a ciliated protozoan that was readily consumed by the prymnesiophyte. Gene expression of *P. parvum* was significantly affected by the presence of other microbial taxa. Transcription data suggested that *P. parvum* obtained organic carbon from both the bacteria and ciliate populations. The data also suggested that the two different prey types provided different nutrients to *P. parvum*, namely organic nitrogen from the ciliate and iron from the bacteria. However, despite providing nutrients, the presence of prey had little positive impact on the growth rate of *P. parvum*, although nutrients released from prey maintained algal population growth longer than in axenic cultures.

## Materials and methods

### Organisms and cultures

*Prymnesium parvum* strain UOBS-LP0109 (Texoma1) was isolated from Lake Texoma, Oklahoma, USA. It was subsequently made axenic by micropipetting single cells through rinses of sterile medium, and then cultured in sterile L1 medium without silica (https://ncma.bigelow.org). The bacterivorous ciliate, *Uronema marina*, was isolated from Buzzards Bay, Massachusetts and maintained on its attendant bacterial flora by periodic subculturing into filtered sterile seawater containing 0.01–0.001% yeast extract.

### Transcriptomics experiment

Three treatments were performed for transcriptomic analysis: axenic *P. parvum*, non-axenic *P. parvum* with its attendant bacterial flora (hereafter called bacterized treatment), and an axenic culture of *P. parvum* to which an aliquot of the bacterized ciliate culture was added (hereafter called ciliate treatment). The latter treatment was prepared by inoculating an axenic *P. parvum* culture in mid-late exponential phase with an aliquot of the ciliate culture in early stationary phase (~50,000 ciliates· ml^−1^) to yield an alga:ciliate ratio of 100:1. Ciliate biomass strongly dominated the ciliate culture at the time of innoculation, although attendant bacteria were also present at ~10^5^ · ml^−1^ in the combined culture. Cultures were examined using a dissecting microscope every 30–40 min following addition of the ciliates to determine when ciliates were eliminated by the alga. All ciliates were rapidly killed (<6 h) after addition of ciliates to the *P. parvum* culture, and the cultures were harvested as soon as no live ciliates remained.

All three treatments were grown in 1–2 L volumes in 2800 ml Pyrex glass Fernbach flasks in L1 medium minus silica at 18 ppt salinity and 18°C. Cultures were incubated at an irradiance of ~300 μE·m^−2^s^−1^, measured using a QSL-100 sensor with QSP-170 deckbox (Biospherical Instruments Inc.). Cultures were incubated on a 12:12 h light:dark cycle.

### Growth experiment

The effect of the presence or absence of prey on the growth of *P. parvum* was established in batch cultures of the alga. All cultures used in growth experiments were grown under the same conditions as the transcriptome experiment except that they were in ~100 mL volumes. The same three treatments of *P. parvum* that were used for transcriptomic analysis were examined. All treatments were conducted in duplicate. On day 16, cells from a ciliate culture were added to an axenic *P. parvum* culture at a ratio of 100:1 (alga:ciliate). Growth of *P. parvum* was monitored by performing direct microscopic cell counts on samples periodically removed from each flask over a 28-day period. Growth rates in different treatments were compared using One-Way ANOVA test.

### RNA extraction and cDNA production

All samples were harvested during mid-exponential growth phase in the middle of the 12 h light cycle. Cultures were spun down in an Eppendorf 5810R centrifuge using the A-4-62 rotor at 3200 rcf for 15 min at 15°C. The supernatant was carefully decanted, and 1–2 ml of TRI reagent (Ambion) was added to the pellet and vortexed until the pellet fully dissolved. Homogenates were then either processed immediately using Ribopure kit (Ambion), or stored at −80°C for later processing. Eluted RNA was treated with DNase (Sigma) to remove DNA contamination. Samples were cleaned and concentrated using RNA Clean and Concentrator-25 (Zymo Research). RNA was quantified using a Qubit 2.0 Fluorometer (Invitrogen) and run on an E-gel iBase with E-gel Gel EX 1% (Invitrogen) to check for nucleic acid quality.

RNA samples were sent to the National Center for Genome Resources for processing and sequencing. RNA was quantified again at the sequencing center using Invitrogen Qubit Q32855, and RNA quality was assessed using the Agilent 2100 Bioanalyzer. cDNA libraries were made from 2 μg RNA using Illumina's TruSeq RNA Sample Preparation Kit. The average insert size of each library ranged from 250 to 350 bp. Libraries were sequenced on an Illumina HiSeq 2000 which generated 2 × 50 bp (paired-end) reads. The original sequence data are publicly available from NCBI Sequence Read Archive under accession number SRA166613 and sample IDs MMETSP0006_2, MMETSP0815, and MMETSP0008_2, for axenic, bacterized and ciliate treatment, respectively.

### Sequencing and bioinformatic analyses

Bioinformatic analysis procedures were adapted and developed from guidelines established by the Marine Microbial Eukaryote Transcriptome Sequencing Project (https://www.marinemicroeukaryotes.org) (Keeling et al., 2014). Sequences of all treatments were first checked for quality using the FASTX toolkit (http://hannonlab.cshl.edu/fastx_toolkit/index.html) with options “-p 80 -q 20.”

An assembled transcriptome of *P. parvum* was then generated as a reference for downstream alignment processes. All sequences of the ciliate treatment were excluded from the assembly to ensure that the *P. parvum* assembled transcriptome was not contaminated with ciliate sequences that might have remained after ciliates were killed by the alga. Although the bacterized treatment and ciliate treatment also contained bacteria, the sequence data from those treatments should have been reasonably free of bacterial sequences because RNA was polyA-selected in our protocol for all treatments. Sequences from the axenic and bacterized treatments were combined and assembled *de novo* using a hybrid approach containing both a de Bruijn graph and an overlap-based algorithm method. Sequences were first assembled using ABySS v. 1.3.2 (Simpson et al., 2009) at four different k-mer settings of 19, 25, 31, and 37. The resulting four assemblies were merged using Trans-ABySS v. 1.4.4 (Robertson et al., 2010). Redundant contigs were removed from the assembly using CD-Hit-EST v. 4.5.7 (Li and Godzik, 2006) with option “-c 0.99.” The remaining contigs were then further assembled using the overlap-based program CAP3 (Huang and Madan, 1999) with options “-p 99 -o 50 −k 0.” Scaffolding of the resulting contigs was inferred using ABySS with default parameters (Simpson et al., 2009). The GapCloser v. 1.12 application of the SOAPdenovo (Luo et al., 2012) was employed to try to fill in gaps in the assembly. Scaffolds were broken into contigs where gaps remained unfilled. Contigs shorter than 150 bp were discarded. CD-Hit-EST was used again to remove redundant contigs.

All contigs were searched against SILVA database (Quast et al., 2013) using BLAST to identify and remove rRNA sequences. Protein coding genes with at least 150 bp were predicted from the remaining contigs using ESTscan v. 3.0.3 (Iseli et al., 1999). Genes were annotated using an evalue cutoff of 1e-5 based on a variety of database searches including HMMER3 v. 3.1b1 (http://hmmer.janelia.org) searches against Pfam (http://pfam.sanger.ac.uk) and Tigrfam (http://www.jcvi.org/cgi-bin/tigrfams/index.cgi) databases. A BLAST search against NCBI nr database was also carried out using the same e-value cutoff. Genes without hits to Pfam or Tigrfam were annotated based on their best hits in the NCBI nr database. KEGG and Gene Ontology annotations were also obtained using KEGG Automatic Annotation Server (http://www.genome.jp/tools/kaas/) and Blast2GO v. 2.5 (Conesa et al., 2005), respectively, to provide additional functional understanding of the genes. Annotations of some genes of interest were edited after manual inspection of all the information available.

Sequences from each of the three datasets were aligned back to the assembled transcriptome using BWA v. 0.6.1-r104 (Li and Durbin, 2009). Read pairs that were aligned to genes correctly (i.e., paired reads aligned to the same gene, on the opposite strand, with fragment size of ~200 bp) were counted using a custom PERL script. Statistical analyses of the read counts of each gene were carried out using the “exact test” function of edgeR v. 3.6.2 (Robinson et al., 2010) with common dispersion set at 0.1. Pairwise comparisons between all three treatments were carried out. *P*-values were adjusted to false discovery rate using p.adjust in R software v. 3.1.0 (Benjamini and Hochberg, 1995). Only genes with adjusted *p*-values smaller than 0.05 were deemed as having significantly different expression levels between treatments. Normalized expression levels in the form of FPKM (Fragments per kilobase exon per million fragments aligned) values were calculated. Expression levels in bacterized and ciliate treatments relative to those in axenic treatment were calculated and plotted in figures.

## Results

### Growth experiment

**Figure 1:**
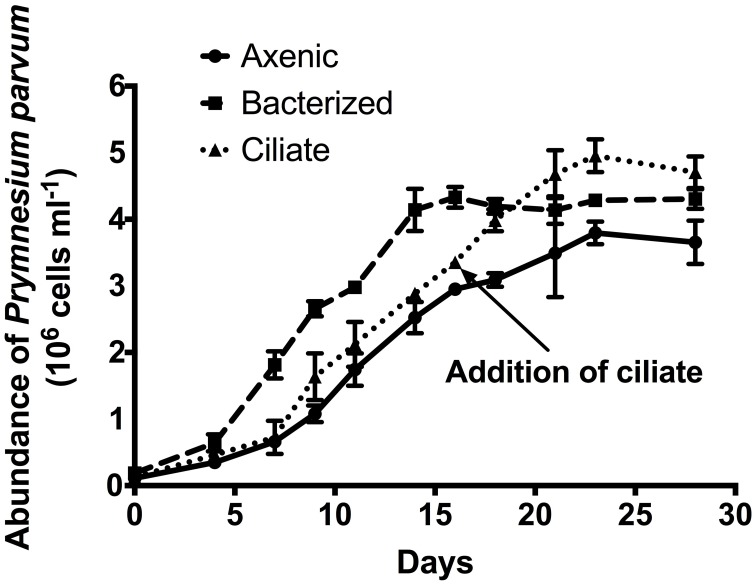
**Growth of the prymnesiophyte, *Prymnesium parvum*, in axenic culture (circles, solid line), in non-axenic culture with attendant bacterial flora (squares, dashed line), and in axenic culture with the addition of ciliates at late exponential phase (triangles, dotted line)**. Arrow indicates Day 16 when ciliates were added to the axenic *P. parvum* culture. In the transcriptome experiment, cells were harvested from axenic and bacterized treatment just prior to the addition of ciliates, while cells were harvested from ciliate treatment ~6 h after the addition of ciliates. Symbols and error bars represent mean ± standard deviation of two replicates.

A growth experiment was conducted to determine whether the presence of prey (bacteria or ciliates) had a significant effect on the growth rate or overall yield of *P. parvum* (Figure 1). Growth rates of the alga were not significantly different (*p* = 0.20, ANOVA) among the three treatments in early exponential phase (0.36 ± 0.02, 0.42 ± 0.01, 0.38 ± 0.04 d^−1^ during the first 9 days of the experiment for axenic, bacterized, and ciliate treatment, respectively. Axenic and ciliate treatments did not differ during that period). The addition of ciliates at late exponential phase of the alga on day 16 allowed *P. parvum* to continue growing (0.12 ± 0.05 d^−1^) while cultures without ciliates exhibited negligible growth (0.03 ± 0.01 d^−1^). *P. parvum* in the bacterized treatment reached the maximum abundance of 4.28 ± 0.03 × 10^6^ cells mL^−1^, which is slightly higher than that of the axenic treatment (3.80 ± 0.17 × 10^6^ cells mL^−1^). The addition of ciliates also increased the maximum abundance of *P. parvum* to (4.95 ± 0.25 × 10^6^ cells mL^−1^).

### Overview of the transcriptome

The assembled transcriptome of *P. parvum* contained 55,984 contigs and 43,058 genes or partial genes, totaling 47.42 million base pairs (Table 1). Most of the physiologically necessary genes were found in the transcriptome (Table S1). For example, the transcriptome contained all genes necessary for glycolysis, the TCA cycle, nitrogen metabolism, nucleotide biosynthesis and almost all genes required for the biosynthesis of all 20 amino acids. Based on this information, it is reasonable to assume that the transcriptome had enough sequencing depth to cover most of the transcribed genes. Consistent with previous experiments (McLaughlin, 1958; Rahat and Reich, 1963), the *P. parvum* transcriptome contained no genes for the synthesis of thiamine and cobalamin but did contain most of the genes required for the synthesis of other vitamins.

**Table 1:** **Summary of three transcriptome datasets of *Prymnesium parvum***.

	**Axenic**	**Bacterized**	**Ciliate**
No. of reads^a^	19,277,859 pairs	33,615,796 pairs	17,560,521 pairs
Size of assembled transcriptome^b^	55,984 contigs; 47.42 Mbp; N50 = 1281 bp; 43,058 predicted genes; average 800 bp		N/A
Reads mapped back to transcriptiome	77.6%	75.8%	69.3%
Reads mapped back to predicted genes	61.5%	62.0%	61.0%

a After quality filtering (see Materials and Methods for quality filtering standards).

b The transcriptome of axenic and bacterized treatments were combined to generate an assembly. The ciliate treatment was not included in the assembly to exclude ciliate sequences from the assembled transcriptome. Only contigs and genes with at least 150 bp were retained for further analysis.

**Figure 2:**
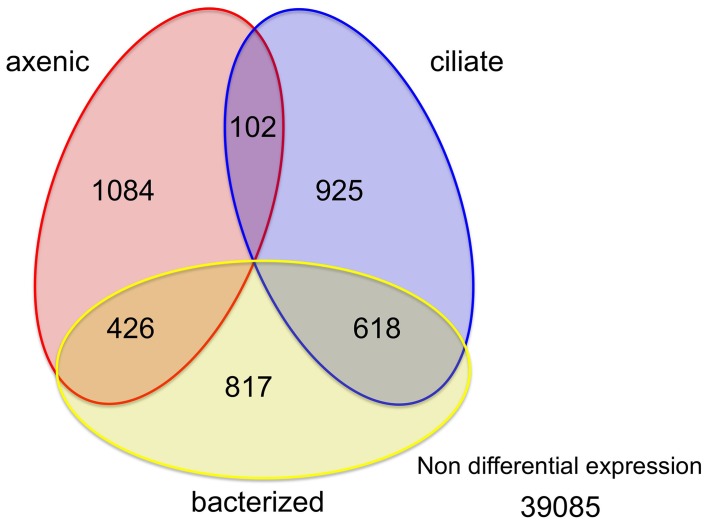
**Distribution of genes according to changes in their expression levels among three treatments**. Numbers in each area indicate numbers of genes whose expression levels were higher in that treatment compared to the other two or in two treatments compared to the third.

Among the 43,058 genes identified in this study, only a small percentage (9.2% or 3972 genes) had significantly different expression levels between at least a pair of treatments (Figure 2). Similar numbers of genes (1600~1900) had higher expression levels in each of the three treatments (1612, 1861, and 1645 genes in the axenic, bacteria and ciliate treatments, respectively; Figure 2). The overlap among those sets of genes, however, was quite interesting. A total of 618 genes, or 33 and 38% of all genes with elevated expression levels in the bacterized and ciliate treatments, respectively, had higher expression levels in both treatments compared to the axenic treatment. In comparison, 426 genes were shared between the axenic and bacterized treatments, and only 102 genes were shared between the axenic and ciliate treatments, in terms of genes with higher expression levels compared to the third treatment (Figure 2).

**Figure 3:**
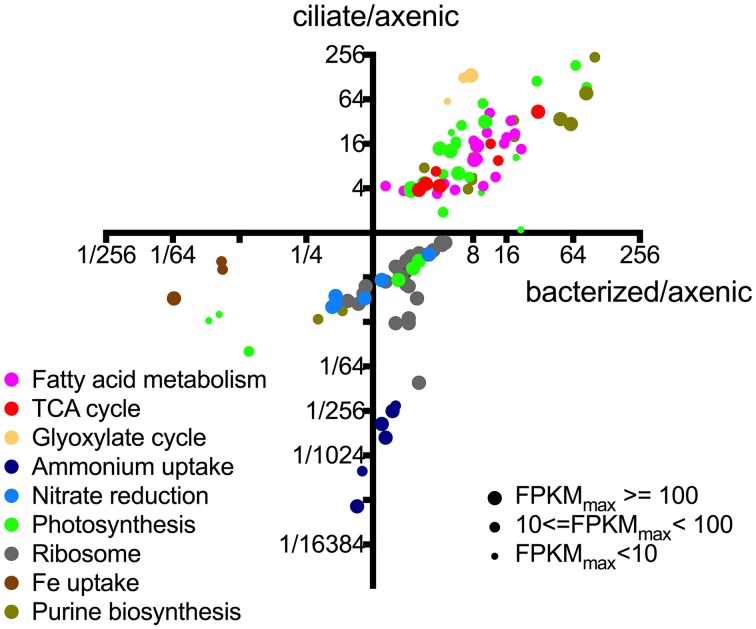
**Relative expression levels of individual genes of *P. parvum* within different metabolic pathways or functions**. Each dot represents an individual gene. X-axis values indicate expression levels in bacterized treatment relative to those in axenic treatment (FPKM_bacterized_/FPKM_axenic_). Y-axis values indicate expression levels in ciliate treatment relative to those in axenic treatment (FPKM_ciliate_/FPKM_axenic_). Sizes of the dots are proportional to the number of reads assigned to the genes. Not all genes within certain pathways or functions are shown, only those that have differential expression between at least a pair of treatments. These functions and pathways with significantly differential gene expression were plotted in Figures 3–**5** to further show consistent patterns within each function/pathway that are highlighted in our discussion of core metabolism of *P. parvum.* For list of genes and their read counts, FPKM values, and relative expression levels, refer to Table S2.

A more detailed evaluation of differential gene expression and function with respect to roles in *P. parvum* showed that most (>50%) of its genes had no matches or functional annotations to existing databases, owing to the relative scarcity of protistan sequence data (Caron et al., 2009). Nevertheless, we were able to infer interesting patterns from changes in the transcriptional patterns of those genes with putative functions (Figure 3). Genes belonging to the same pathway or metabolic function had very similar transcriptional patterns among the treatments. For example, all differential expressed genes associated with fatty acid metabolism had higher expression levels in the presence of either prey type and formed a cluster in the upper right quadrant of Figure 3, while genes associated with iron uptake clustered together in the lower left quadrant (i.e., the latter genes had lower expression levels in the presence of both prey). Figure 3 also illustrates that genes associated with different metabolic functions often demonstrated different transcriptional patterns (as noted above). While changes in transcription do not necessarily equate with protein concentrations or enzymatic activities, the transcriptional patterns observed in this study provided insights into the relationship between *P. parvum* and the presence of potential prey, and different prey types.

### Carbon metabolism

**Figure 4:**
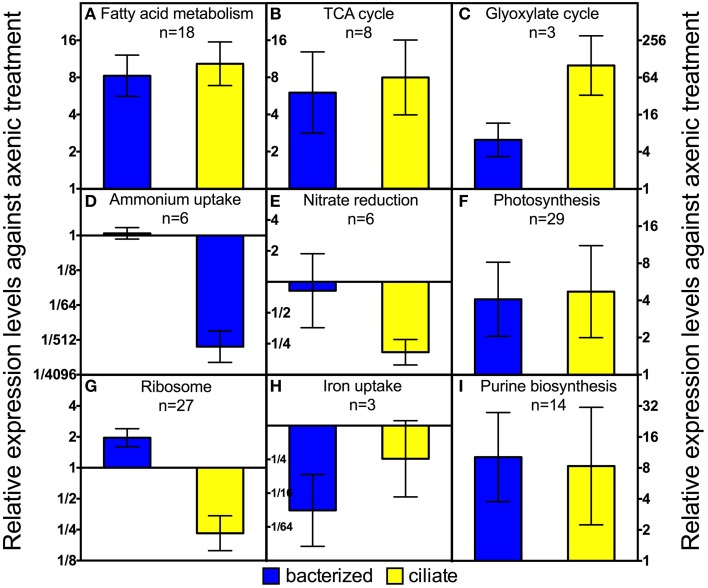
**Average expression levels among different groups of genes of *P. parvum* in bacterized and ciliate treatment relative to those in axenic treatment**. Geometric means ± 95% confidence intervals are shown for relative expression levels against the axenic treatment of genes of each pathway or function. For list of genes and their read counts, FPKM values, and relative expression levels, please refer to Table S2.

Genes involved in several carbon metabolism pathways had higher expression levels in both treatments with prey, compared to the axenic treatment. Several copies of genes of every step in the fatty acid oxidation pathway, including acyl-CoA dehydrogenase, enoyl-CoA hydratase, 3-hydroxyacyl-CoA dehydrogenase, and acetyl-CoA C-acetyltransferase, had ~9-fold higher expression levels when prey, either bacteria or ciliates, were present (Figures 3, 4A). Two genes encoding an electron transfer flavoprotein gene and an electron transfer flavoprotein ubiquinone oxidoreductase that also play a role in fatty acid oxidation (Watmough and Frerman, 2010) had similar transcription patterns (Table S2).

Fatty acid oxidation produces a large quantity of acetyl-CoA, which can be used in two different pathways in *P. parvum* mitochondria: complete oxidation through the TCA cycle or transformation to succinate through the glyoxylate cycle, with the former pathway being catabolic and the latter being anabolic. As expected, genes involved in both pathways had higher expression levels as a result of increased influx of acetyl-CoA when prey were available (Figures 4B,C). Genes responsible for almost every step of the TCA cycle were regulated in strikingly similar patterns as those of fatty acid oxidation (Figure 4B). Expression levels of two genes specifically involved in the glyoxylate cycle, malate synthase and isocitrate lyase (the rest of the pathway overlaps with the TCA cycle) increased ~6-fold in the bacterized treatment relative to the axenic one, and increased ~100-fold when ciliates were present (Figure 4C).

**Figure 5:**
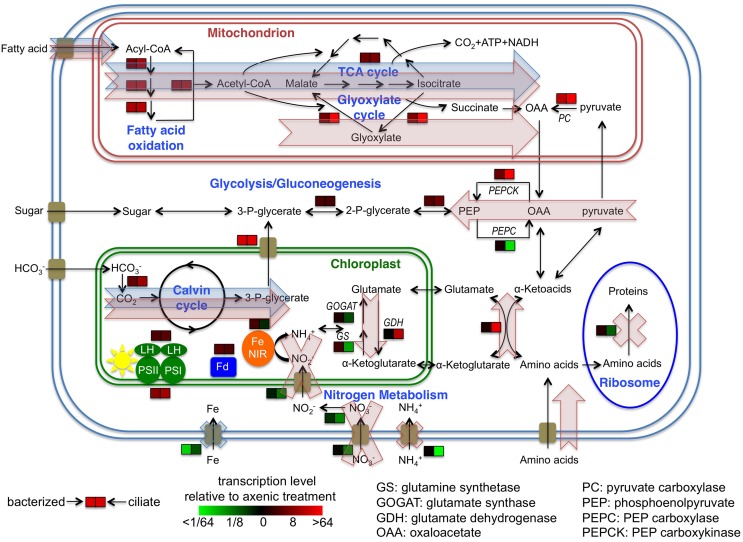
**Cellular overview of gene transcription of *Prymnesium parvum* under three treatments and hypothetical model of metabolic activities**. Transcription levels of select genes, pathways or functions in bacterized and ciliate treatments relative to axenic treatment are shown. Blue shaded arrows and crosses indicate proposed model of metabolic flows and suppression of functions of *P. parvum*, respectively, in response to the presence of bacteria in the bacterized treatment. Red shaded arrows and crosses indicate the same flows and suppressions in response to the presence of ciliates in the ciliate treatment. Cellular localizations of proteins are based on knowledge of homologs in other protists or *in silico* predictions.

Expression patterns of glycolysis/gluconeogenesis genes were inconsistent among themselves. However, genes involved in the connection between glycolysis/gluconeogenesis and other carbon pathways had interesting expression patterns. Genes encoding pyruvate carboxylase and PEP carboxykinase had slightly higher expression levels in the bacterized treatment, and even higher levels in the ciliate treatment. These two proteins convert pyruvate and TCA cycle metabolites through oxaloacetate to PEP, and are generally regarded as gluconeogenic enzymes. Conversely, PEP carboxylase, which operates in the opposite direction of PEP carboxykinase, decreased significantly in its transcription level in the ciliate treatment (Figure 5, Table S2).

### Nitrogen metabolism

Transcription data suggested dramatic changes in modes of nitrogen acquisition in response to the addition of prey and particularly to the presence of the ciliates. First, expression levels of ammonium uptake genes drastically decreased in the ciliate treatment, compared to the other two treatments (Figure 4D). Those genes include several copies of an ammonium transporter and two periplasmic L-amino acid oxidases. The latter two genes were similar to a homolog in the green algal species, *Chlamydomonas reinhardtii*, that has been shown to oxidize extracellular amino acids to release ammonium (Vallon et al., 1993). Similar although less dramatic transcriptional changes were observed for nitrate reduction genes. Nitrate transporter, nitrate reductase, ferredoxin-nitrite reductase and several nitrite transporters had ~6-fold lower expression levels in the ciliate treatment relative to the axenic or bacterized treatment (Figure 4E).

The balance and interconversion between α-ketoglutarate and glutamate is central to nitrogen metabolism, especially amino acid metabolism, as the pair provide the amino group to most if not all amino acids during their biosynthesis, and accept the amino group when they are metabolized. Expression levels of glutamine synthetase (GS) and glutamine oxoglutarate aminotransferase (GOGAT), which converts α-ketoglutarate to glutamate, decreased significantly in the ciliate treatment. On the other hand, expression levels of glutamate dehydrogenase (GDH), which catalyzes the opposite reaction, increased significantly (Figure 5, Table S2). Meanwhile, an aminotransferase gene, which catalyzes the exchange of amino groups between α-ketoglutarate/glutamate and other amino acids/α-ketoacids, had significantly higher expression levels in the ciliate treatment (Figure 5, Table S2).

### Photosynthesis

A large number of genes involved in photosynthesis, such as most photosynthetic reaction center genes and ribulose-bisphosphate carboxylase genes are encoded by the chloroplast genome. Their mRNAs were not retained in the polyA selection process in our protocol, and their sequences were largely undetected in our datasets. However, we were still able to obtain transcriptional patterns of many chromosomal genes involved in photosynthesis or CO_2_ fixation (Figure 4F). Four genes encoding photosystem I or II proteins were detected in the transcriptome, and all of them had higher expression levels when prey were present. The transcriptome also included many copies of chlorophyll *a*/*b* binding proteins, some of which were among the most actively transcribed genes. Of these, 20 genes were differentially expressed, and 17 of those had higher expression levels in the presence of prey. Two genes encoding plant type ferredoxin, the major electron acceptor from photosystem I, increased their expression levels in the presence of prey. Three genes encoding carbonic anhydrases, which supply CO_2_ to the Calvin cycle, had similar expression patterns. Two other genes encoding phosphoglycerate transporters that presumably transport the end products of the Calvin cycle outside of chloroplast (Neuhaus and Wagner, 2000) had similar expression patterns (Figure 5, Table S2).

### Other notable pathways and functions

Twenty-seven genes encoding ribosomal proteins exhibited similar transcriptional patterns. On average, their expression levels decreased ~4-fold in the ciliate treatment compared to the axenic treatment (Figure 4G). The same genes had slightly higher expression levels (~2 fold) in the bacterized treatment compared to the axenic treatment.

Genes encoding three proteins involved in iron uptake were regulated similarly. They include the low iron inducible periplasmic protein FEA1 and two high affinity iron permeases FTR1. FEA1 has been shown to be one of the major proteins excreted by iron deficient *Chlamydomonas reinhardtii* (Allen et al., 2007). FEA1 may also facilitate iron uptake when expressed heterologously in yeast and plants (Narayanan et al., 2011). FTR1 is part of a high affinity iron uptake system consisting of a permease and an oxidase (Stearman et al., 1996). The transcriptional levels of these three genes were ~30-fold lower in the bacterized treatment than in the axenic treatment. Those levels also decreased, although to a lesser degree (~8-fold) in the ciliate treatment (Figure 4H).

Expression levels of 14 genes involving in purine biosynthesis increased in both bacterized and ciliate treatments compared to axenic treatment (Figure 4I). Most of the 15 genes encode proteins involved in the early, generic part of the purine biosynthesis pathway. Therefore, it was unclear whether there was a targeted increase in adenine or guanine biosynthesis. In comparison, pyrimidine biosynthesis genes did not change significantly among the three treatments.

Genes responsible for the biosynthesis of prymnesins are not yet fully characterized. However, since the two identified prymnesins are polyketides (Igarashi et al., 1996), it has been speculated that polyketide synthases play an important role in the synthesis of prymnesins (Manning and La Claire, 2010). Eleven different genes annotated as polyketide synthase were found in the transcriptome. Among them, only one had differential expression (slightly higher in the ciliate treatment), the other 10 were not differentially expressed in any of the treatments. Homologs of the genes previously studied by qPCR (Freitag et al., 2011) were among those not differentially expressed.

Three different phosphate transporters were differentially expressed. However, the pattern of their expression among the three treatments was not consistent. An ABC-type and a Pho4 family phosphate transporter both had higher expression levels (~4-fold) in the ciliate treatment. A sodium-dependent phosphate transporter had higher expression levels in the bacterized treatment (~4-fold).

## Discussion

### Comparison with previous gene expression studies

The size of the *P. parvum* transcriptome is comparable to other protistan transcriptomes in the Marine Microbial Eukaryote Transcriptome Sequencing Project (https://www.marinemicroeukaryotes.org), the transcriptomes of other prymnesiophytes (Koid et al., 2014), and protistan genomes in general (Caron et al., 2009). The transcriptome was also compared to EST data previously obtained for *P. parvum*. Out of 6286 EST sequences generated for *P. parvum* strain UTEX no. 2797 (La Claire, 2006), 5626 (89.5%) had a homolog in the transcriptome of clone UOBS-LP0109 obtained in this study, with average nucleotide sequence identity of 98.9%. Out of 17,153 EST sequences generated for *P. parvum* strain RL10 (Beszteri et al., 2012), 14,271 (83.2%) had a homolog in this transcriptome, with average nucleotide sequence identity of 95.8%. The numbers of shared genes were probably slight underestimates because of the limitations of the *de novo* short read assembly. *P. parvum* strain UOBS-LP0109 shares a large numbers of genes with both other strains, but was more closely related to strain UTEX no. 2797 than strain RL10 in terms of both percentage of shared genes and sequence similarity. This finding is not surprising considering the proximity of the sources of strain UOBS-LP0109 (Lake Texoma, on the border of Oklahoma and Texas) and strain UTEX no. 2797 (Texas). Many of the most frequently-encountered ESTs in the study of strain UTEX no. 2797 (La Claire, 2006), such as multiple chlorophyll-binding proteins, ABC-type phosphate transporter, and phosphoenolpyruvate carboxykinase, were also among the genes with the most read pairs.

### Nutrient uptake from prey

Numerous studies have documented the ability of *P. parvum* to kill or prey on a variety organisms, ranging from bacteria to phototrophic and heterotrophic protists, zooplankton and even fish (Tillmann, 2003; Southard et al., 2010; Remmel and Hambright, 2012). It has been reported that heterotrophic nutrition by this toxic, mixotrophic alga may provide a substantial percentage of the macronutrients and carbon required for growth (Carvalho and Granéli, 2010). However, the specific contribution to algal nutrition provided by consumed prey remains unclear, and there is virtually no information on how *P. parvum* might respond at the cellular or molecular level to the availability of prey. We evaluated changes in gene transcription of the alga in response to two different prey assemblages: a mixed bacterial assemblage, or one consisting of largely a ciliated protist with a background assemblage of bacteria. We realize the lack of replication in our transcriptome study and therefore have been purposefully conservative in our interpretation of the data. In many cases, different genes involved in the same pathway had similar transcription patterns (Figure 3), thereby providing a level of confidence to their collective transcription patterns. Inference from the data was only drawn in those cases, and was never based on the transcription pattern of a single gene. We also recognize and acknowledge the complexity of the ciliate treatment in that many factors could have affected gene transcription of *P. parvum* leading to difficulties in attributing *P. parvum* response to a specific factor. Those factors include ciliate cells, bacterial cells, nutrients from the ciliate culture, and nutrients released as a consequence of ciliates consuming bacteria. We assume that *P. parvum* gene transcription changes in the ciliate treatment were likely the result of its predation on ciliates because ciliate biomass greatly outweighed bacterial biomass in that treatment, but dissolved substances released by the ciliates may also have elicited responses from the alga. Regardless, our results clearly indicated that *P. parvum* killed prey and acquired nutrients in their presence, and also provided insights into the response of specific metabolic processes affected by the presence of other microorganisms.

The transcriptomic data obtained in this study indicate some shared changes in gene expression to the presence of bacteria or ciliates (relative to the alga grown axenically), as well as some changes that were specific to these two types of prey assemblages. For carbon metabolism, expression levels of genes involved in fatty acid metabolism and the TCA cycle were higher in both treatments with prey (Figures 4A,B). This finding indicates that *P. parvum* probably used fatty acids from both prey types. Changes in expression levels of genes involved in the glyoxylate cycle, pyruvate carboxylase, PEP carboxykinase, and PEP carboxylase were much more dramatic in the ciliate treatment than in the bacterized treatment (Figure 5). This implies that organic carbon from prey was used in different ways in those two treatments. Specifically, it seemed that more fatty acids were used for biomass production and less for energy production in the ciliate treatment (Figure 5).

In nitrogen metabolism, significant changes in gene expression were only observed in the ciliate treatment. The presence of ciliates led to dramatic decreases in expression levels of genes involved in inorganic nitrogen uptake, especially ammonium uptake (Figures 3, 4D). On the other hand, the transcription patterns of GS, GOGAT, GDH and an aminotransferase gene indicate increased catabolism of amino acids in the ciliate treatment (Figure 5). Collectively, these data suggests that amino acids, presumably obtained either directly from the ciliate or mediated by the ciliate (i.e., perhaps as a consequence of ciliate predation on co-occurring bacteria), were metabolized in *P. parvum*. Nitrogen needs of *P. parvum* were likely satisfied by the influx of organic nitrogen, which led to the suppression of inorganic nitrogen uptake. In all, these data imply that ciliates might be a very effective nitrogen source for *P. parvum* in nature. Ciliates have been shown to be an important source of nitrogen for other mixotrophic protists in nitrogen-limited environments (Bockstahler and Coats, 1993; Smalley and Coats, 2002). This hypothesis is also consistent with previous findings that have reported greater toxicity of *P. parvum* under nitrogen or phosphate limitation (Johansson and Granéli, 1999; Granéli and Johansson, 2003; Hambright et al., 2014), and suppression of toxicity when high doses of ammonium are provided (Grover et al., 2007, 2013).

Expression levels of genes involved in iron uptake decreased sharply in the presence of bacteria, and to a lesser degree in the ciliate treatment (Figure 4H). Both prey assemblages were likely sources of iron for *P. parvum* in our experiment, although it would appear that bacteria alone were more effective in making iron accessible than the ciliate. This finding is consistent with the varied and complex relationships known to occur between phytoplankton and co-occurring bacteria (Amin et al., 2012). Maranger et al. (1998) experimentally demonstrated that the ingestion of bacteria by a different mixotrophic phytoflagellate, *Ochromonas* sp., provided iron for cellular growth of the alga. We speculate that the digestion of ingested prey by *P. parvum* may meet the iron requirement for growth of the alga and thereby reduce the need for iron uptake and transport. Alternatively, iron availability may be increased in the presence of other microbes, obviating a requirement for phagotrophy by *P. parvum*. For example, predation on bacteria by heterotrophic protists has been shown to relieve iron stress of co-occurring algae (Barbeau et al., 1996). Most previous studies of *P. parvum* have focused on the importance of nitrogen and phosphate contained in prey biomass as a factor in the toxicity of the alga, since N:P ratio has a direct impact on the toxicity of *P. parvum* (Granéli et al., 2012). Our results broaden these findings to include the possible importance of iron to the metabolism of *P. parvum*. The bacterial assemblage in our culture was part of the native bacterial assemblage occurring during a *P. parvum* bloom. It would be interesting to determine whether *P. parvum* has a preference among different bacteria as a means of acquiring iron.

### Contribution of predation to the growth of *p. parvum*

The ciliate employed in the study, *Uronema marina*, was readily killed when introduced into cultures of *P. parvum* at high abundance. The process involved immobilization and attachment of several algal cells to each ciliate, as has been described previously (Tillmann, 2003). Bacterivory has also been reported for *P. parvum* (Nygaard and Tobiesen, 1993), although ingestion of bacteria was not confirmed in our study. It has been speculated that prey capture and digestion by *P. parvum* provide substantial sources of major nutrients (N, P) or organic carbon for energy and growth (Carvalho and Granéli, 2010; Granéli et al., 2012). Our transcriptional data supported this hypothesis, for nitrogen and carbon.

Many mixotrophic protists grow faster when prey are available compared to axenic growth (Sanders, 2011). Surprisingly, our transcriptional data and our direct measurements of growth of the alga indicated that *P. parvum* did not grow faster despite its demonstrable predatory behavior. Protein synthesis at ribosomes is a huge component of cell growth, therefore ribosome synthesis could at least in part reflect cell growth. It has been proposed that ribosome synthesis levels could be a good measure for growth (Rudra and Warner, 2004). The expression levels of genes encoding *P. parvum* ribosomal proteins increased only slightly in the bacterized treatment compared to levels observed for the axenic treatment (Figure 4G). However, *P. parvum* did not grow significantly faster in the presence of bacteria (Figure 1). On the other hand, the expression levels of these genes decreased in the ciliate treatment (Figure 4G), implying *P. parvum* synthesized fewer ribosomes. Although the addition of ciliates in the late exponential phase maintained growth of *P. parvum*, that growth rate was slower than that of an axenic culture in the early-mid exponential phase (*p* < 0.001, ANOVA, Figure 1). The treatments with prey did support slightly higher yields of the alga relative to the axenic *P. parvum* culture. This outcome is presumably a result of the additional nutrients available from the prey or the culture medium of the prey. Overall, our findings are consistent with a previous study in which *P. parvum* fed *Rhodomonas salina* did not grow faster than a monoculture of *P. parvum*, even under nutrient deficient conditions (Carvalho and Granéli, 2010).

One possible explanation for the lack of increased growth rate in the presence of prey is the cost of making heterotrophic cellular machinery when prey become available. However, we did not observe an initial decrease followed by later recovery in growth rate that would be expected if such hypothesis were true. Additionally, killing of the ciliate cells started almost instantaneously when they were added to the *P. parvum* culture, suggesting *P. parvum* was already toxic and predatory when growing axenically. Therefore, a more likely explanation is that *P. parvum* is critically dependent on some aspect of phototrophy, and that phototrophy presents a bottleneck controlling growth rate, at least under the specific growth conditions employed in this study. A photosynthetic bottleneck is implied by the observation that *P. parvum* cannot grow or survive in the dark even when prey are available at high abundances (Carvalho and Granéli, 2010; Brutemark and Granéli, 2011). The only known exception to this rule is that *P. parvum* can grow very slowly in the dark when supplied with high concentrations of certain organic compounds (Rahat and Jahn, 1965). However, the concentrations of organic compounds required for its growth in the dark are much higher than concentrations that would be experienced in nature. The mechanism and metabolic details of such growth are unknown.

Carvalho and Granéli (2010) suggested that photosynthesis and phagotrophy in *P. parvum* might supplement each other, employing “co-metabolism” for growth. Our results do not directly support this hypothesis, since the presence of prey in our study did not enhance growth rate *per se* beyond that enabled by phototrophic growth. However, one observation in our study was at least partially consistent with their hypothesis. A number of genes involved in photosynthesis, although far from complete and conclusive, increased their expression levels in response to prey despite the fact that there was no difference in light conditions during the experiment. The presence of prey may have relieved some of the cellular processes required to obtain major nutrient elements (e.g., N), as evidenced by changes in gene expression among our experimental treatments. As a consequence, more resources might have been made available for making the photosynthetic apparatus. Alternatively, prey may have provided some micronutrient(s) that support photosynthesis. For example, increased availability of iron may have had a positive effect on photosynthesis since the photosynthetic apparatus includes many proteins and cofactors that contain Fe-S clusters.

It was unclear from our transcriptomic data where or how nutrients acquired by *P. parvum* from its heterotrophic activities were used, since growth rate was not noticeably enhanced. One possibility is storage, possibly in carbohydrates. Some of our data supported this hypothesis as expression levels of some gluconeogenic genes increased in treatments with prey, especially in the ciliate treatment (Figure 5). However, genes involved in starch biosynthesis were not significantly regulated. Other forms of storage are also possible, although there were no transcriptional data indicating which form. If this hypothesis is true, it means that *P. parvum* may be acquiring nutrients from prey and storing them for growth when conditions are amenable for photosynthesis. This is consistent with the observation that *P. parvum* took up nitrogen and phosphorus from *Myrionecta rubra* but did not grow in the dark (Brutemark and Granéli, 2011).

Our study investigated expression of a wide range of genes in the mixotrophic protist *P. parvum* in the presence or absence of two different prey assemblages. Our gene expression data clearly indicated that *P. parvum* obtained a variety of nutrients from its prey including organic carbon in the form of fatty acids from both the bacteria and the ciliate, organic nitrogen in the form of amino acids from the ciliate, and iron from the bacteria. Despite the nutrient gains, *P. parvum* did not grow faster, implying that its growth is ultimately controlled by its photosynthetic activity, at least in the cases of tested prey types. Our study revealed transcriptional behaviors of *P. parvum* in response to prey availability, and has led to several testable hypotheses relating to interactions between *P. parvum* and its prey. These results provide the necessary foundation for future molecular studies regarding the contribution of prey for growth and maintenance of this ecologically and economically important organism.

### Conflict of interest statement

The authors declare that the research was conducted in the absence of any commercial or financial relationships that could be construed as a potential conflict of interest.
