# Supplementary material for: Gene expression in the mixotrophic prymnesiophyte, Prymnesium parvum, responds to prey availability
Source: Front Microbiol. 2015 Apr 20;6:319. doi: 10.3389/fmicb.2015.00319 (PMC4403553; doi:10.3389/fmicb.2015.00319)
Supplement: Supplementary file 1 [file Table1.DOC]

Table S1. Summary of presence/absence of necessary genes in central metabolic pathways in *P. parvum* transcriptome according to KEGG annotations.

| Function/Pathway | Gene content summary |
| --- | --- |
| Glycolysis/Gluconeogenesis | All necessary genes detected |
| TCA cycle | All necessary genes detected |
| Fatty acid metabolism | All necessary genes detected |
| Purine/Pyrimidine biosynthesis/metabolism | All necessary genes detected |
| Ala/Asp/Glu biosynthesis/metabolism | All necessary genes detected |
| Gly/Ser/Thr biosynthesis/metabolism | All necessary genes detected |
| Cys/Met biosynthesis/metabolism | *mccB* not detected |
| Val/Leu/Ile biosynthesis/metabolism | All necessary genes detected |
| Lysine biosynthesis/metabolism | All necessary genes detected |
| Arg/Pro biosynthesis/metabolism | All necessary genes detected |
| Histidine biosynthesis/metabolism | *hisB* not detected |
| Phe/Tyr/Trp biosynthesis/metabolism | *pheC*, *tyrB*, *tyrA2*, *trpF* not detected |
