# Supplementary material for: Gene expression in the mixotrophic prymnesiophyte, Prymnesium parvum, responds to prey availability
Source: Front Microbiol. 2015 Apr 20;6:319. doi: 10.3389/fmicb.2015.00319 (PMC4403553; doi:10.3389/fmicb.2015.00319)
Supplement: Supplementary file 2 [file Table2.DOC]

Table S2. Expression levels of genes mentioned in this study in the form of FPKM (Fragment per kilobase exon per million fragments mapped) with the original fragment counts in the parentheses. Relative expression levels of those genes in bacterized and ciliate treatment compared with axenic treatment are also listed.

| Gene ID | Expression levels: FPKM(original counts) | | | Relative expression levels compared with axenic ^a^ | | Annotation (KEGG orthology number) |
| --- | --- | --- | --- | --- | --- | --- |
|  | axenic | bacterized | ciliate | bacterized | ciliate |  |
| **Fatty acid metabolism** | | | | | | |
| 1007 | 5.7(59) | 56.6(1028) | 24.7(231) | 9.9 | 4.3 | enoyl-CoA isomerase (K13239) |
| 2075 | 5.6(82) | 71.5(1849) | 31.6(421) | 12.8 | 5.7 | acetyl-CoA C-acetyltransferase (K00626) |
| 3338 | 0.5(12) | 5.1(239) | 18.9(454) | 11.3 | 41.8 | fatty acid oxidation complex, alpha subunit (K07515) |
| 3500 | 26.7(265) | 217.5(3802) | 257.4(2314) | 8.2 | 9.7 | electron transfer flavoprotein domain |
| 4193 | 8.0(211) | 70.4(3269) | 79.5(1898) | 8.8 | 9.9 | electron transfer flavoprotein-ubiquinone oxidoreductase |
| 4408 | 6.6(97) | 53.4(1382) | 115.9(1542) | 8.1 | 17.6 | acyl-CoA dehydrogenase (K00249) |
| 4981 | 1.0(10) | 19.0(336) | 22.0(200) | 19.1 | 22.1 | enoyl-CoA hydratase/isomerase family (K01661) |
| 6741 | 8.6(142) | 75.5(2181) | 131.0(1946) | 8.7 | 15.1 | acetyl-CoA C-acetyltransferase (K07509) |
| 8143 | 11.6(274) | 63.7(2638) | 44.7(953) | 5.5 | 3.8 | acyl-CoA synthetase (K01897) |
| 8203 | 6.6(111) | 25.3(747) | 22.2(337) | 3.8 | 3.4 | acetyl-CoA C-acetyltransferase (K00626) |
| 13125 | 2.5(40) | 54.5(1535) | 34.0(493) | 21.8 | 13.6 | fatty acid oxidation complex, alpha subunit FadB (K07516) |
| 44176 | 4.6(69) | 74.3(1950) | 90.8(1226) | 16.1 | 19.6 | 3-hydroxyacyl-CoA dehydrogenase PaaC (K00022) |
| 54554 | 21.6(337) | 94.6(2602) | 100.2(1417) | 4.4 | 4.6 | acyl-CoA dehydrogenase, N-terminal domain (K00248) |
| 56247 | 14.0(229) | 26.1(752) | 51.1(757) | 1.9 | 3.7 | acyl-CoA dehydrogenase (K00252) |
| 57144 | 9.4(133) | 11.9(298) | 40.4(519) | 1.3 | 4.3 | short/branched chain acyl-CoA dehydrogenase (K09478) |
| 60784 | 1.6(42) | 17.2(788) | 36.6(862) | 10.7 | 22.7 | fatty acid oxidation complex, alpha subunit (K07515) |
| 65356 | 0.9(6) | 14.3(161) | 15.2(88) | 15.3 | 16.2 | enoyl-CoA hydratase/3-hydroxyacyl-CoA dehydrogenase (K10527) |
| 84443 | 4.0(56) | 70.2(1719) | 131.9(1662) | 17.5 | 32.8 | acetyl-CoA C-acetyltransferase (K07508) |
| **TCA cycle** | | | | | | |
| 6455 | 79.3(997) | 319.9(7073) | 341.2(3881) | 4.0 | 4.3 | malate dehydrogenase, NAD-dependent (K00026) |
| 8676 | 65.4(961) | 193.2(4991) | 301.1(4000) | 3.0 | 4.6 | succinate-CoA ligase, beta subunit (K01900) |
| 46954 | 4.8(93) | 55.2(1885) | 76.5(1344) | 11.5 | 16.0 | Fumarate hydratase (Fumerase) (K01676) |
| 59391 | 89.4(1978) | 230.0(8948) | 339.9(6801) | 2.6 | 3.8 | succinate dehydrogenase, flavoprotein subunit (K00234) |
| 59778 | 15.5(555) | 57.6(3628) | 104.9(3397) | 3.7 | 6.8 | oxoglutarate dehydrogenase (succinyl-transferring), E1 component (K00164) |
| 60620 | 1.8(10) | 5.6(54) | 8.8(44) | 3.1 | 4.9 | malate dehydrogenase, NAD-dependent (K00026) |
| 109650_1 | 5.5(145) | 169.3(7900) | 238.3(5720) | 31.0 | 43.6 | monomeric isocitrate dehydrogenase |
| 110049_2 | 7.0(241) | 95.4(5742) | 66.7(2066) | 13.5 | 9.5 | aconitate hydratase 2 (K01682) |
| **Glyoxylate cycle** | | | | | | |
| 1333 | 20.6(476) | 159.4(6479) | 2768.9(57900) | 7.7 | 134.4 | isocitrate lyase (K01637) |
| 57667 | 0.8(23) | 5.1(269) | 95.9(2609) | 6.6 | 125.4 | malate synthase (K01638) |
| 60981 | 0.1(3) | 0.5(25) | 5.8(163) | 4.7 | 60.0 | malate synthase (K01638) |
| **Ammonium uptake** | | | | | | |
| 7718 | 233.1(5482) | 167.9(6943) | 0(0) | 0.72 | 0.0002 | L-amino-acid oxidase |
| 10673 | 16.0(238) | 25.6(671) | 0(0) | 1.6 | 0.0046 | ammonium transporter |
| 14620 | 418.6(6722) | 610.1(17235) | 1.7(24) | 1.5 | 0.0039 | ammonium transporter |
| 18108 | 84.1(1735) | 67.6(2456) | 0.1(1) | 0.80 | 0.0006 | periplasmic L-amino acid oxidase, catalytic subunit |
| 110256_1 | 89.3(426) | 107.7(904) | 0.2(1) | 1.2 | 0.0026 | ammonium transporter |
| 110256_2 | 331.8(638) | 434.6(1470) | 0(0) | 1.3 | 0.0017 | ammonium transporter |
| **Nitrate reduction** | | | | | | |
| 14500 | 148.7(3288) | 478.3(18606) | 76.0(1521) | 3.2 | 0.51 | nitrite reductase (K00366) |
| 84400 | 374.1(11444) | 171.0(9202) | 40.8(1130) | 0.46 | 0.11 | nitrate reductase (K10534) |
| 99993 | 262.3(551) | 121.2(448) | 37.9(72) | 0.46 | 0.14 | formate/nitrite transporter |
| 105139 | 504.1(1113) | 217.3(844) | 50.1(100) | 0.43 | 0.10 | formate/nitrite transporter |
| 108982 | 174.4(385) | 215.3(836) | 39.5(79) | 1.2 | 0.23 | formate/nitrite transporter |
| 109209_2 | 1132(25092) | 954.2(37198) | 147.7(2961) | 0.84 | 0.13 | nitrate transporter (K02575) |
| **Photosynthesis** | | | | | | |
| 329_1 | 2.9(12) | 18.1(133) | 82.0(309) | 6.3 | 28.5 | chlorophyll A-B binding protein |
| 329_2 | 1.1(4) | 9.6(59) | 17.4(55) | 8.4 | 15.2 | chlorophyll A-B binding protein |
| 2890 | 58.7(487) | 4.5(65) | 1.5(11) | 0.076 | 0.025 | chlorophyll A-B binding protein |
| 2905 | 28.7(94) | 123.5(712) | 55.7(165) | 4.3 | 1.9 | ferredoxin [2Fe-2S] (K02639) |
| 7722 | 138.8(1020) | 397.1(5133) | 657.6(4372) | 2.9 | 4.7 | Photosystem II Pbs27 (K08902) |
| 9789 | 1.4(9) | 7.6(88) | 23.4(139) | 5.6 | 17.1 | chlorophyll A-B binding protein |
| 11533 | 4.1(17) | 0(0) | 0(0) | 0.033 | 0.065 | chlorophyll A-B binding protein |
| 13182_1 | 0.1(2) | 9.8(238) | 26.6(333) | 67.6 | 184.0 | carbonic anhydrase (K01673) |
| 15115 | 5.9(50) | 43.7(654) | 33.4(257) | 7.4 | 5.7 | photosystem II Psb28 (K08903) |
| 15859 | 406.5(2562) | 906.3(10048) | 1614.6(9208) | 2.2 | 4.0 | chlorophyll A-B binding protein |
| 16421 | 448.8(847) | 1027.1(3410) | 147.6(252) | 2.3 | 0.33 | chlorophyll A-B binding protein |
| 17064 | 164.4(324) | 425.3(1474) | 68.4(122) | 2.6 | 0.42 | chlorophyll A-B binding protein |
| 17668 | 0.4(2) | 12.2(106) | 45.6(204) | 30.1 | 112.7 | chlorophyll A-B binding protein |
| 18397 | 84.9(508) | 343.1(3611) | 1168.3(6324) | 4.0 | 13.8 | chlorophyll A-B binding protein |
| 18420_1 | 0.4(2) | 8.0(69) | 4.3(19) | 19.6 | 10.5 | chlorophyll A-B binding protein |
| 18983 | 2.1(7) | 20.8(121) | 118.8(356) | 9.8 | 56.2 | chlorophyll A-B binding protein |
| 41092 | 12.0(596) | 45.3(3964) | 51.7(2329) | 3.8 | 4.3 | photosystem I P700 chlorophyll a apoprotein A1 (K02689) |
| 56904 | 11.5(85) | 49.6(646) | 71.2(477) | 4.3 | 6.2 | ferredoxin [2Fe-2S] (K02639) |
| 59596_1 | 0.3(2) | 28.5(298) | 31.2(168) | 84.7 | 92.8 | photosystem II psbU |
| 59747 | 6.9(43) | 38.5(420) | 108.8(610) | 5.6 | 15.7 | chlorophyll A-B binding protein |
| 79183 | 6.4(14) | 0(0) | 0(0) | 0.041 | 0.079 | chlorophyll A-B binding protein |
| 82969 | 1.1(7) | 10.2(117) | 3.7(22) | 9.5 | 3.5 | chlorophyll A-B binding protein |
| 86696 | 0.2(3) | 1.1(27) | 5.0(62) | 5.1 | 22.8 | carbonic anhydrase (K01673) |
| 102302 | 420.3(868) | 702.7(2553) | 94.7(177) | 1.7 | 0.23 | chlorophyll A-B binding protein |
| 105556 | 81.5(1814) | 409.6(16034) | 1037.6(20895) | 5.0 | 12.7 | carbonic anhydrase (K01673) |
| 106580 | 117.0(579) | 686.6(5978) | 745.8(3340) | 5.9 | 6.4 | chlorophyll A-B binding protein |
| 109160_1 | 0.2(1) | 4.4(38) | 0(0) | 21.6 | 1.1 | chlorophyll A-B binding protein |
| 109241_1 | 78.7(687) | 811.2(12450) | 2506.4(19785) | 10.3 | 31.8 | chlorophyll A-B binding protein |
| 109379_2 | 1282.0(8902) | 2802(34226) | 4737.3(29764) | 2.2 | 3.7 | chlorophyll A-B binding protein |
| **Ribosome** | | | | | | |
| 127 | 363.3(1902) | 1631(15021) | 268.4(1271) | 4.5 | 0.74 | small subunit ribosomal protein S20e (K02969) |
| 4133 | 180.5(752) | 359.9(2638) | 75.3(284) | 2.0 | 0.42 | small subunit ribosomal protein S15e (K02958) |
| 5945 | 246.3(2283) | 1002(16347) | 181.5(1522) | 4.1 | 0.74 | small subunit ribosomal protein S5e (K02989) |
| 7961 | 1039.6(1925) | 1337.3(4356) | 225.6(378) | 1.3 | 0.22 | large subunit ribosomal protein L39e (K02924) |
| 9644 | 126.9(1392) | 443.4(8554) | 73.6(730) | 3.5 | 0.58 | small subunit ribosomal protein SAe (K02998) |
| 15367 | 549.0(3226) | 1405(14522) | 292.3(1554) | 2.6 | 0.53 | small subunit ribosomal protein S11e (K02949) |
| 15636 | 153.4(306) | 277.9(975) | 39.9(72) | 1.8 | 0.26 | ribosomal protein P1 family |
| 16001 | 915.7(4239) | 679.3(5532) | 97.2(407) | 0.74 | 0.11 | large subunit ribosomal protein L7/L12 (K02935) |
| 16024_1 | 50.3(440) | 107.0(1646) | 3.5(28) | 2.1 | 0.07 | large subunit ribosomal protein L19e (K02885) |
| 17732_1 | 205.4(790) | 167.5(1133) | 31.0(108) | 0.82 | 0.15 | large subunit ribosomal protein L29 |
| 52152 | 54.3(118) | 143.2(547) | 0.5(1) | 2.6 | 0.0094 | large subunit ribosomal protein LP0 (K02941) |
| 55970 | 81.8(271) | 164.6(959) | 18.4(55) | 2.0 | 0.2 | small subunit ribosomal protein S16e (K02960) |
| 56963 | 201.9(1582) | 173.4(2390) | 38.7(274) | 0.86 | 0.19 | large subunit ribosomal protein L13 (K02871) |
| 60177 | 480.6(1369) | 285.4(1430) | 58.6(151) | 0.59 | 0.12 | large subunit ribosomal protein L24 (K02895) |
| 83098 | 42.9(133) | 76.2(415) | 14.6(41) | 1.8 | 0.34 | large subunit ribosomal protein L31e (K02910) |
| 84153 | 538.3(4313) | 1288(18166) | 254.2(1843) | 2.4 | 0.47 | small subunit ribosomal protein S8e (K02995) |
| 85654 | 395.1(4244) | 850.6(16073) | 190.6(1853) | 2.2 | 0.48 | small subunit ribosomal protein S7e (K02993) |
| 85736 | 54.7(154) | 95.6(473) | 14.5(37) | 1.7 | 0.27 | small subunit ribosomal protein S27Ae (K02977) |
| 89136 | 120.9(241) | 298.2(1046) | 16.1(29) | 2.5 | 0.13 | large subunit ribosomal protein L17/L22 family |
| 90861 | 560.1(2613) | 1381(11339) | 279.1(1178) | 2.5 | 0.50 | large subunit ribosomal protein L35e (K02918) |
| 108472 | 445.0(1891) | 733.0(5479) | 157.1(604) | 1.6 | 0.35 | small subunit ribosomal protein S27e (K02978) |
| 109156_1 | 567.6(4103) | 1767(22472) | 288.4(1886) | 3.1 | 0.51 | large subunit ribosomal protein L7Ae (K02936) |
| 109156_2 | 206.6(780) | 869.1(5771) | 141.7(484) | 4.2 | 0.69 | large subunit ribosomal protein L7Ae (K02936) |
| 109806_1 | 236.4(724) | 491.6(2648) | 14.8(41) | 2.1 | 0.06 | large subunit ribosomal protein L23Ae ((K02893) |
| 109806_2 | 174.4(441) | 279.0(1241) | 10.9(25) | 1.6 | 0.06 | large subunit ribosomal protein L23Ae (K02893) |
| 109812_2 | 34.0(92) | 68.7(327) | 9.4(23) | 2.0 | 0.28 | large subunit ribosomal protein L23e (K02894) |
| 110227_2 | 119.2(314) | 250.7(1162) | 23.1(55) | 2.1 | 0.19 | large subunit ribosomal protein L17e (K02880) |
| **Iron uptake** | | | | | | |
| 8794 | 79.2(649) | 3.5(50) | 25.6(190) | 0.044 | 0.32 | iron permease FTR1 |
| 9684 | 36.9(318) | 1.6(24) | 15.2(119) | 0.043 | 0.41 | iron permease FTR1 |
| 76086 | 418.9(880) | 6.5(24) | 52.6(100) | 0.016 | 0.13 | low iron-inducible periplasmic protein FEA1 |
| **Purine biosynthesis** | | | | | | |
| 1591 | 2.4(55) | 17.6(701) | 9.4(193) | 7.2 | 3.9 | ADP-ribose pyrophosphatase (K13988) |
| 4146 | 5.8(84) | 109.2(2800) | 114.7(1513) | 18.9 | 19.9 | phosphoribosylaminoimidazole-succinocarboxamide synthase (K01923) |
| 4184 | 3.9(46) | 73.8(1516) | 131.9(1394) | 18.7 | 33.5 | phosphoribosylaminoimidazole-succinocarboxamide synthase (K01923) |
| 9181 | 0.8(14) | 6.6(192) | 9.3(139) | 7.8 | 11.0 | amidophosphoribosyltransferase (K09580) |
| 16103 | 4.9(84) | 296.9(9019) | 144.5(2258) | 61.0 | 29.7 | adenylosuccinate lyase (K01756) |
| 17493 | 5.1(96) | 14.7(483) | 39.0(660) | 2.9 | 7.6 | adenylosuccinate synthase (K01939) |
| 18836 | 13.0(72) | 101.7(990) | 67.1(336) | 7.8 | 5.2 | amidophosphoribosyltransferase (K00764) |
| 42278 | 22.0(265) | 11.7(248) | 1.9(21) | 0.53 | 0.088 | ribonucleoside-diphosphate reductase subunit M2 (K10808) |
| 55482 | 2.4(111) | 198.9(16417) | 182.7(7758) | 84.1 | 77.2 | phosphoribosylformylglycinamidine synthase (K01952) |
| 85903 | 0.7(15) | 5.4(214) | 3.9(79) | 8.1 | 5.8 | GMP synthase (glutamine-hydrolysing) (K01951) |
| 106468 | 24.1(163) | 7.8(93) | 1.6(10) | 0.32 | 0.068 | ADP-ribose pyrophosphatase (K01515) |
| 108281 | 0.2(4) | 19.0(708) | 44.8(859) | 100.6 | 237.3 | phosphoribosylaminoimidazolecarboxamide formyltransferase (K00602) |
| 108445 | 7.0(33) | 59.0(488) | 75.2(320) | 8.4 | 10.7 | amidophosphoribosyltransferase (K00764) |
| 109215_2 | 5.0(182) | 243.9(15707) | 171.8(5689) | 49.1 | 34.5 | phosphoribosylformylglycinamidine cyclo-ligase (K11788) |
| **Other carbon metabolism** | | | | | | |
| 572 | 0.3(5) | 49.1(1363) | 122.3(1746) | 155.0 | 385.9 | phosphoglycerate transporter family protein |
| 3801 | 17.1(315) | 61.8(1998) | 24.2(402) | 3.6 | 1.4 | pyruvate kinase (K00873) |
| 6687 | 1.6(72) | 36.3(2807) | 93.1(3699) | 22.2 | 56.8 | pyruvate carboxylase (K01958) |
| 13564 | 0.1(1) | 11.6(403) | 16.3(293) | 229.1 | 323.8 | pyruvate kinase (K00873) |
| 14648 | 610.9(2045) | 553.0(3256) | 70.0(212) | 0.91 | 0.11 | Phosphoglycerate kinase (K00927) |
| 15061 | 54.4(1964) | 82.6(5242) | 1.3(44) | 1.5 | 0.025 | Phosphoenolpyruvate carboxylase (K01595) |
| 56146 | 0.0(1) | 6.4(372) | 8.9(264) | 211.5 | 291.8 | pyruvate kinase (k00873) |
| 59594 | 30.0(639) | 168.2(6301) | 2177.1(41947) | 5.6 | 72.6 | phosphoenolpyruvate carboxykinase (ATP) (K01610) |
| 82481 | 24.4(564) | 98.3(4004) | 59.6(1248) | 4.0 | 2.4 | Phosphoglycerate kinase (K00927) |
| 108363 | 3.6(54) | 73.7(1957) | 19.5(267) | 20.6 | 5.5 | phosphoglycerate transporter family protein |
| 109623_3 | 94.9(490) | 114.1(1036) | 21.8(102) | 1.2 | 0.23 | glyceraldehyde 3-phosphate dehydrogenase, NAD binding domain (K00134) |
| 109623_5 | 1108.2(5525) | 1236(10847) | 242.8(1095) | 1.1 | 0.22 | glyceraldehyde-3-phosphate dehydrogenase, type I (K00134) |
| **Other nitrogen metabolism** | | | | | | |
| 3169 | 85.9(4901) | 144.1(14459) | 31.5(1628) | 1.7 | 0.37 | ferredoxin-dependent glutamate synthase (K00264) |
| 9858 | 4.2(111) | 40.3(1886) | 0.9(21) | 9.7 | 0.21 | glutamine synthetase (K01915) |
| 18657 | 160.8(2279) | 409.1(10200) | 41.8(536) | 2.5 | 0.26 | glutamine synthetase (K01915) |
| 26267 | 2.6(17) | 4.2(49) | 110.4(665) | 1.6 | 43.2 | aminotransferase (K14455) |
| 55820 | 26.9(718) | 25.8(1212) | 4.1(98) | 1.0 | 0.15 | glutamine synthetase (K01915) |
| 59677_1 | 7.4(262) | 7.2(446) | 144.9(4647) | 1.0 | 19.6 | glutamate dehydrogenase (K15371) |

^a^Numbers of read pairs were changed from 0 to 1 when calculating relative expression levels in order to avoid division by zero.
